# Harnessing the potential of spatial multiomics: a timely opportunity

**DOI:** 10.1038/s41392-023-01507-3

**Published:** 2023-06-12

**Authors:** Xuexin Li

**Affiliations:** Department of Medical Biochemistry and Biophysics, Karolinska Institutet, Solna, Sweden

**Keywords:** Assay systems, Systems biology

In a recent publication in Nature,^1^ Zhang et al. have developed spatial ATAC-RNA-seq and spatial CUT&Tag-RNA-seq as innovative tools that enable the high-resolution mapping of epigenetic and transcriptomic features in cells from frozen tissue sections. By applying these techniques to mouse embryos and brains, the authors discovered unexpected interrelationships between epigenetic regulation and gene expression in various brain regions, leading to new insights on spatial epigenetic activation, differentiation, and gene regulation in tissue structure, with significant implications for understanding gene regulation during development and disease.

The paper outlines the detailed methodology of the spatial ATAC-RNA-seq and spatial CUT&Tag-RNA-seq techniques. The procedures entail the use of a Tn5 transposition complex to attach a DNA adaptor with a universal ligation linker to accessible genomic DNA loci in formaldehyde-fixed frozen tissue sections. Subsequently, a biotinylated DNA adaptor containing a poly-T sequence is used to initiate reverse transcription within the same tissue. Spatial barcodes are introduced using a microfluidic channel array chip, generating a two-dimensional grid of spatially barcoded tissue pixels. After reverse crosslinking, the barcoded complementary DNA and genomic DNA fragments are released, enriched, and separated for next-generation sequencing. Spatial CUT&Tag-RNA-seq involves applying an antibody specific to histone modifications on the tissue section and using a protein A-tethered Tn5-DNA complex to execute CUT&Tag.

Next, the research team utilized Spatial ATAC-RNA-seq analysis to study mouse embryos on embryonic day 13 (E13). By analyzing chromatin accessibility and transcriptomics data, they identified marker genes specific to different cell clusters and successfully differentiated various organs of mouse embryos. Joint clustering of the spatial ATAC-seq and RNA-seq data allowed for improved spatial cell type mapping, identifying a new neuronal cluster that single modalities could not resolve. The team also investigated the correlation between accessible peaks and expressed genes in the tissue context, identifying distinct signals at predicted enhancers for certain genes. The study focused on the differentiation trajectory from radial glia to postmitotic premature neurons, utilizing spatial ATAC-RNA-seq technology to investigate the spatiotemporal relationship between chromatin accessibility and gene expression during embryonic development. The results revealed gene regulatory mechanisms and spatiotemporal dynamics in tissue development, highlighting the potential use of the technology in the study of tissue development.

The researchers conducted a follow-up investigation on the mouse juvenile brain on postnatal day 22 (P22) and identified major cell types. They observed a general alignment between chromatin accessibility and transcriptome in defining cell identities. By conducting joint spatial multi-omics analysis, the team detected 21,417 significant peak-to-gene linkages between regulatory elements and target genes. They discovered unexpected connections between epigenetic regulation and gene expression in different regions of the developing brain. Several affected genes encoded transcription factors related to neural development and were present from the embryonic stage. However, some marker genes with open chromatin accessibility, such as Sox10, Sox2, Neurod6, Pax6, and Notch1, were either not expressed or expressed at low levels. This suggests the possibility of epigenetic memory of these genes during brain development, rather than transcriptional regulation.

Furthermore, Zhang et al. performed spatial CUT&Tag-RNA-seq to co-profile the transcriptome and three histone modifications (H3K27me3, H3K27ac, and H3K4me3) in the P22 mouse brain. They identified clusters for specific histone modifications and RNA that corresponded with anatomical annotation by Nissl staining. By integrating spatial CUT&Tag and single-cell CUT&Tag data, they confirmed that the epigenetic states observed in their data were consistent with the single-cell data for H3K27me3, H3K27ac, and H3K4me3. The study used chromatin silencing score (CSS) and gene activity score (GAS) to predict gene expression for different histone modifications, revealing region-specific marker gene modifications and corresponding RNA signatures. The findings highlight the importance of utilizing CUT&Tag and RNA-seq simultaneously in the same tissue section for more precise identification of cell types or states based on epigenetic profiles.

Additionally, the team demonstrated the utility of spatial ATAC-RNA-seq in analyzing an adult brain and discovered that combining the two methods could provide new insights into gene regulation mechanisms. They found that the gene PROX1, which is important for defining granule neuron identity, had modest chromatin accessibility despite being highly expressed in the granule cell layer. This suggests that mature granule cells may not require an active open chromatin state for gene expression. The study underscores the potential of spatial cosequencing of the epigenome and transcriptome in uncovering dynamic gene regulation mechanisms.

Spatial ATAC-RNA-seq and spatial CUT&Tag-RNA-seq are emerging technologies that allow for the measurement of both the epigenome and transcriptome on tissue sections, providing insights into the spatial correlation and regulatory mechanisms of genetic information at various levels. With near-single-cell resolution and the ability to cover the entire genome, these technologies have been demonstrated as useful tools for defining cell states and depicting dynamic landscapes in mouse embryos, juvenile brains, and adult human brains. These technologies offer a powerful tool for studying spatial epigenetic regulation in life sciences and biomedical research.

In 2022, *Nature* recognized Spatial Omics technology as one of the top seven technologies to watch,^2^ acknowledging its potential to revolutionize our understanding of complex tissue microenvironments and dynamic biological processes. Despite the emergence of various spatial omics technologies in recent years (Fig. 1),^3^ several formidable challenges still need to be addressed before their full potential can be realized.^4,5^ One of the most critical challenges is to achieve high spatial resolution while preserving the sensitivity and specificity of the molecular assays. This requires the development of novel experimental and computational techniques capable of accurately detecting signals from individual cells or subcellular structures in intricate tissue microenvironments. Furthermore, the integration of spatial omics data with other types of molecular and clinical data is a major challenge that necessitates the development of advanced computational methodologies and the standardization of experimental protocols and data analysis pipelines. The cost of equipment and reagents required for Spatial Omics analysis is another significant obstacle that can restrict access to this technology for researchers with limited financial resources. Moreover, interpreting Spatial Omics data in the context of clinical outcomes is challenging due to the intricacy of tissue microenvironments and dynamic biological processes. Therefore, close collaboration between computational biologists, biostatisticians, clinicians, and translational researchers is necessary to surmount these challenges and unlock the full potential of Spatial Omics technology in basic and clinical research.

**Fig. 1:**
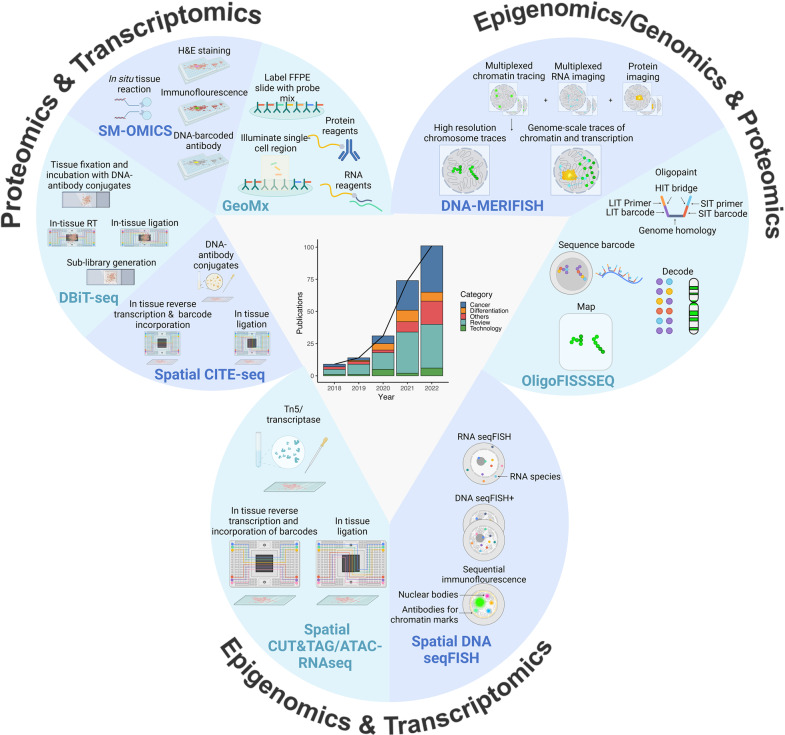
Representative Technologies and Publications in Spatial Multiomics: A Revisit of the Last Five Years. The line graph illustrates the number of publications on single-cell spatial multi-omics over the past five years, categorized into five groups based on relevant keywords. Meanwhile, the accompanying bar plot displays the publication count for each category per year, sourced from Pubmed. The figure also showcases representative technologies and important procedures, with the three categories based on the omics combination. For single-cell spatial proteomics and transcriptomics, the technologies presented include spatial CITE-seq (Cellular Indexing of Transcriptomes and Epitopes by Sequencing), DBiT (Deterministic barcoding in tissue for spatial omics sequencing), SM-OMICS (Spatial Multi-Omics), and GEOMx (GeoMx DSP). For single-cell spatial epigenomics and transcriptomics, the figure introduces spatial CUT&TAG/ATAC RNAseq (Cleavage Under Targets & Tagmentation/Assay for Transposase-Accessible Chromatin using sequencing) and spatial DNA seqFISH (DNA sequential Fluorescence In Situ Hybridization) as representative technologies. Lastly, for single-cell spatial epigenomics/genomics and transcriptomics, the figure showcases spatial DNA-MERFISH (DNA-Multiplexed error-robust fluorescence in situ hybridization) and Oligo FISSEQ (Oligo fluorescent in situ sequencing) as representative technologies. The figure also includes several abbreviations, such as FFPE for “formalin-fixed paraffin-embedded”, H&E for “Hematoxylin and eosin stain”, RT for “reverse transcription”, LIT for “ligation-based interrogation of targets”, SIT for “sequencing by synthesis to effect synthesis-based interrogation of targets”, and HIT for “hybridization-based interrogation of targets”. Figure created with BioRender.com

Despite the difficulties, the potential of Spatial Omics technology in diverse fields such as tumor research, immunology, pathology research, neuroscience, and developmental biology is vast and capable of transforming the landscape of medical research. Spatial Omics technology has now reached its prime time.

## References

[1] Zhang D (2023). Spatial epigenome-transcriptome co-profiling of mammalian tissues. Nature.

[2] Eisenstein M (2022). Seven technologies to watch in 2022. Nature.

[3] Vandereyken K, Sifrim A, Thienpont B, Voet T (2023). Methods and applications for single-cell and spatial multi-omics. Nat. Rev. Genet..

[4] Miao Z, Humphreys BD, McMahon AP, Kim J (2021). Multi-omics integration in the age of million single-cell data. Nat. Rev. Nephrol..

[5] Park J (2022). Spatial omics technologies at multimodal and single cell/subcellular level. Genome Biol..

